# Duodenal Research

**Published:** 1912-11

**Authors:** 


Duodenal Research—Von Oelfele and Bullinger, *New Yorker Medizinische Monatschrift,* July, 1912. The authors port in extenso on experiments and experiences with their own modification and improvement of Einhorn's duodenal tube; claiming to obtain the duodenal fluids in greater amounts than is possible with other instruments. They make numerous observations of interest to the specialist in this field but draw no practical conclusions.

An especially interesting observation is this: That large amounts of water may pass a stomach containing nutriment with little or no admixture; the food remaining in the fundus, the water apparently passing over it.

